# Characterization of mouse brain microRNAs after infection with cyst-forming *Toxoplasma gondii*

**DOI:** 10.1186/1756-3305-6-154

**Published:** 2013-05-29

**Authors:** Min-Jun Xu, Dong-Hui Zhou, Alasdair J Nisbet, Si-Yang Huang, Yi-Fan Fan, Xing-Quan Zhu

**Affiliations:** 1 State Key Laboratory of Veterinary Etiological Biology, Key Laboratory of Veterinary Parasitology of Gansu Province, Lanzhou Veterinary Research Institute, Chinese Academy of Agricultural Sciences, Lanzhou, Gansu Province, 730046, PR China; 2 Vaccines and Diagnostics, Moredun Research Institute, Pentlands Science Park, Midlothian, Scotland, EH26 0PZ, UK; 3 College of Animal Science and Veterinary Medicine, Heilongjiang Bayi Agricultural University, Daqing, Heilongjiang Province, 163319, PR China; 4 College of Veterinary Medicine, Sichuan Agricultural University, Ya’an, Sichuan Province, 625014, PR China

**Keywords:** Toxoplasma gondii, Toxoplasmosis, MicroRNA (miRNA), Mouse, Brain, Host regulation

## Abstract

**Background:**

The obligate intracellular parasite *Toxoplasma gondii* can interfere with host cell signaling pathways, alter host defense systems and cell cycle control, and establish a chronic infection in the central nervous system. *T. gondii* infection may alter the expression profile of host microRNAs (miRNAs) which have key regulatory functions at the post-transcriptional level.

**Methods:**

Using high-throughput sequencing and real-time quantitative PCR technology, we compared the miRNA expression profiles of uninfected mouse brains with brains from mice at 14 days and 21 days after infection with cyst-forming *T. gondii* (Type II)*.*

**Results:**

A total of 51.30 million raw reads were obtained from all samples and 495 (14d infected mouse sample), 511 (14d sham-infected control), 504 (21d infected mouse sample) and 514 (21d sham-infected control) miRNA candidates identified. Among these, 414 miRNAs were consistent across all the studied groups, 17 were specific to the 14d infected group and 32 were specific to the 21d infected group. In addition, 9 miRNAs were common to both the 14d- and 21d-infected groups. Enrichment analysis for the targets of these miRNAs showed a high percentage of “protein tag” functions. Immune related targets including chemokines, cytokines, growth factors and interleukins were also found.

**Conclusions:**

These results not only showed that the miRNA expression of the host can be changed by the invasion of cyst-forming *T. gondii*, but also indicated that the host attempts to respond using two tactics: marking proteins with “protein tags” and adaptation of immune related systems.

## Background

The apicomplexan parasite *Toxoplasma gondii* has a wide range of hosts and infects warm-blooded animals worldwide [1-5]. It is also one of the most prevalent parasitic infections in humans with nearly one-third of the population worldwide estimated to be infected [6]. Although infections in adults are typically subclinical, *T. gondii* could possibly persists for the life of the host and can have severe, or even fatal, consequences in the case of congenital transfer or in immune-compromised individuals [7,8]. During invasion of the host cells, a set of specialized secretory organelles is used by the parasite, with some of the associated invasion factors interfering with host cell signaling pathways and altering the host defense systems and cell cycle control [9-12]. However, the modulatory mechanisms of these factors are still not well understood.

microRNAs (miRNAs) are 18–22 nucleotides (nt), non-coding, small RNA molecules which regulate gene expression at the post-transcriptional level and are now considered as a key mechanism of gene regulation [13,14]. miRNAs are essential for parasite invasion, development and the ability to respond to environmental and developmental signals, but they also play a role in host cellular differentiation, fitness and defense mechanisms [15-18]. It has previously been reported that *T. gondii* infection can increase the levels of miRNAs in cultured human foreskin fibroblasts (HFFs), such as miR-17 (approximately 92 times) and miR-106b (approximately 25 times) which were known to play crucial roles in cell regulation and were involved in numerous hyperproliferative diseases [19].

*Toxoplasma* can establish a chronic infection in the central nervous system, where it changes from fast-replicating tachyzoites to bradyzoites and evades the host immune system [20,21] and it was recently suggested that this type of infection may result in the initiation and progression of brain cancer by modifying the miRNA expression of human brain cells [22].

To investigate the roles of specific miRNAs in key regulatory functions during *T. gondii* infection, we therefore compared the miRNA expression profiles of mouse brains during infection with *T. gondii*.

## Methods

### Ethics statement

All animals were handled in strict accordance with good animal practice according to the Animal Ethics Procedures and Guidelines of the People’s Republic of China, and the study was approved by the Animal Ethics Committee of Lanzhou Veterinary Research Institute, Chinese Academy of Agricultural Sciences (Permit No. LVRIAEC2010-006).

### Collection of mouse brain tissues

*T. gondii* PRU strain (Type II) was kindly provided by Professor Hai-Zhu Zhang of Department of Parasitology, Xinxiang Medical College, Henan, China. Forty Specific-Pathogen-Free (SPF) grade female Kunming mice (20 mice for control and treatment group respectively), aged from 6 to 8 weeks old, were purchased from the Lanzhou Institute of Biological Products Laboratory Animal Center.

*T. gondii* cysts were harvested from the brains of infected mice, and then counted using an optical microscope. For the “infected” group, each mouse was inoculated intra-gastrically with 10 cysts; and for the control group, sterile physiological saline was used for the sham inoculation. At 14 day (d) and 21 d post infection, mice were euthanized, with the whole brain rapidly collected, washed, and stored at −80 C as described previously [23].

### Total RNA and small RNA isolation

Total RNA was prepared with TRIzol Reagent according to the manufacturer’s protocol (Invitrogen Co. Ltd). For each group, total RNA was extracted from 6 biological replicates and then pooled for small RNA isolation. Small RNA of 20–40 bases was obtained as described previously from 10 μg total RNA using a Novex 15% TBE-Urea gel [24]. The fragments were purified and ligated with 5′ and 3′ adaptors (Illumina), and reverse transcribed with an RT-PCR kit. All the gels and kits for small RNA purification and reverse transcription were purchased from Invitrogen Co. Ltd.

### High-throughput sequencing and bioinformatic analysis

Samples were sequenced using a Solexa sequencer at HuaDa Genomic Co. Ltd. After base-calling, adaptors and reads with low quality were removed. The raw data were searched against the Rfam databases (http://rfam.sanger.ac.uk/) to identify non-coding RNAs, including rRNA, tRNA, snRNA, and snoRNA. Repetitive sequences were identified using RepeatMasker (http://www.repeatmasker.org). The *Mus musculus* genome at UCSC (http://hgdownload.cse.ucsc.edu/goldenPath/mm9/bigZips/chromFa.tar.gz) was downloaded and used as a reference genome against which filtered reads were mapped using SOAP [25]. Precursors of miRNAs were predicted with Mfold (http://www.bioinfo.rpi.edu/applications/mfold). The predicted mature miRNAs were further matched with known *T. gondii* miRNAs deposited in the Sanger miRBase (http://www.mirbase.org/) to remove possible *T. gondii* contamination. Only those miRNAs having standard stem-loop structure and exact locations on the *M. musculus* reference genome were retained to form the miRNA expression profile of each sample. The miRNA profiles of controls and treatments were then compared with each other to identify specific miRNAs in each sample. Targets of specific miRNAs were predicted using TargetScan with the mouse genes selected [26,27]. Functions of the predicted targets were then analyzed with the Gene Ontology (http://www.geneontology.org/) and DAVID databases (http://david.abcc.ncifcrf.gov/).

### Analysis of miRNA expression level

Stem-loop real-time RT-PCR was performed as described previously [28]. The glyceraldehyde-3-phosphate dehydrogenase (Gapdh) of *M. musculus* (NM_008084.2) was used as endogenous control of each reaction and all reactions were performed using an ABI PRISM® 7300 Sequence Detection System. All primers were synthesized by Shenggong Co. Ltd., China and all reactions were carried out in triplicate. The primer pairs for Gapdh quantification were: F: 5′-GGCCTCCAAGGAGTAAGAAA-3′; R: 5′-GCCCCTCCTGTTATTATGG-3′. The cycles were performed as follows: 50°C and 95 C for 2 min respectively, followed by 30 cycles of 95°C for 15 s, 60° for 32 s, and 72 C for 32 s. The relative quantification of each miRNA was calculated using the equation: N = 2^-ΔCt^, ΔCt = Ct_miRNA_-Ct_control_[29].

## Results

### Profile characteristics of short RNAs

A total of 51.30 million raw reads were obtained from all groups. After removing low quality reads and adaptors, 45.53 million high quality reads remained, including 10.48 and 11.04 million reads for the 14 d infected sample and its sham-infected control respectively; and 10.76 and 13.25 million reads for the 21 d infected sample and its sham-infected control respectively.

Length distribution analysis showed that reads of 21-23nt were the most abundant. Repeat analysis revealed that 97 repeat sequence types (representing 5.84% - 7.61% of the unique reads) of which 11 (Ambi, tRNA:1, LINE/L1:0, LINE/L1:1, LTR/MaLR:0, snRNA:1, SINE/B4:1, LTR/MaLR:1, SINE/Alu:1, SINE/B2:0, rRNA:1) were most highly represented with read numbers higher than 1000 (1007–20735 reads). Other non-coding RNAs, including tRNA, rRNA, snRNA and snoRNA, represented a total of 24.43% - 30.65% of the unique reads of the 4 samples; rRNA was the predominant non-coding RNA (13.31-19.04% of the unique reads).

### Global view of miRNA profiles

By mapping against the *M. musculus* genome, we obtained 511 (43 novel + 468 known), 495 (36 + 459), 514 (53 + 461) and 504 (41 + 463) miRNA candidates, with precursors having standard stem-loop structures, from the total RNA of samples of 14d-control, 14d-infection, 21d-control, and 21d-infection, respectively. By matching the miRNA candidates with known *M. musculus* miRNAs deposited in the Sanger miRBase, most of the miRNA candidates were previously known, with only 7.84% (36/459) – 11.50% (53/461) identified as novel.

There were 16 miRNAs with copy numbers higher than 100,000, ranging from 121,582 to 2,233,052 copies. These 16 miRNAs belonged to 7 families; let-7, mir-101, mir-128, mir-107, mir-140, mir-29a and mir-124. The let-7 family, which was the most highly represented in each miRNA profile, had 8 members present from mmu-let-7a to mmu-let-7i. For the mir-101 and mir-128 families, two members were identified in each; mir-101a and mir-101b, and mir-128-1 and mir-128-2.

### Comparison of miRNA profiles

**Figure 1:**
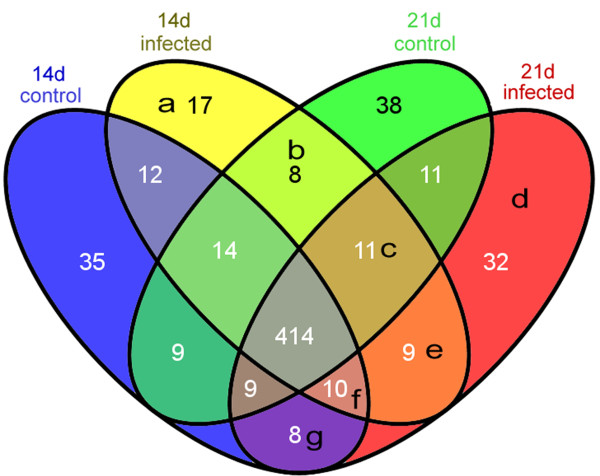
**Comparative miRNA expression profiles of mouse brains infected with *Toxoplasma gondii*.** 14d control and 21d control were brains from control, sham-infected mice; *T. gondii* infected samples were from brains of mice at 14 days and 21 days post infection.

414 miRNAs were common amongst the infected and sham-infected (control) groups (Figure 1). Compared with controls, 45 different miRNAs occurred in the 14d-infected sample (Figure 1a, b, c, e); and 59 different miRNAs occurred in 21d-infected sample (Figure 1d, e, f, g). Among these, 9 miRNAs (Figure 1e) were shared between the two “infected” groups, representing miRNAs, which persist after *T. gondii* infection. Moreover, 17 miRNAs (Figure 1a) were specific to the 14d-infected sample and 32 miRNAs (Figure 1d) were specific to the 21d-infected sample.

### Target prediction and functional analysis

The 17 and 32 specific miRNAs from 14d- and 21d-infected samples, as well as the 9 miRNAs common to both time points post-infection, were used for target prediction (Additional file 1: Table S1). For the 17 specific miRNAs of the 14d-infected sample, 1954 targets were found in total, with 115 on average; the miRNA that possessed the highest number of targets was mmu-miR-9 (1076 targets), with the best matched target being KEL (Kell blood group, metallo-endopeptidase, NM_000420). For the 32 specific miRNAs of the 21d-infected sample, 2407 targets were found in total with 78 on average; the miRNA that possessed the highest number of targets was mmu-miR-694 (888 targets) with the best matched target being CHCHD1 (coiled-coil-helix-coiled-coil-helix domain containing 1, NM_203298). For the 9 miRNAs common to both time points post-infection, the total number of targets was 501 with 63 on average. The miRNA that had the highest number of predicted targets was mmu-miR-5107 (212 targets) and the best matched target was Pafah2 (platelet-activating factor acetylhydrolase 2, NM_133880).

**Figure 2:**
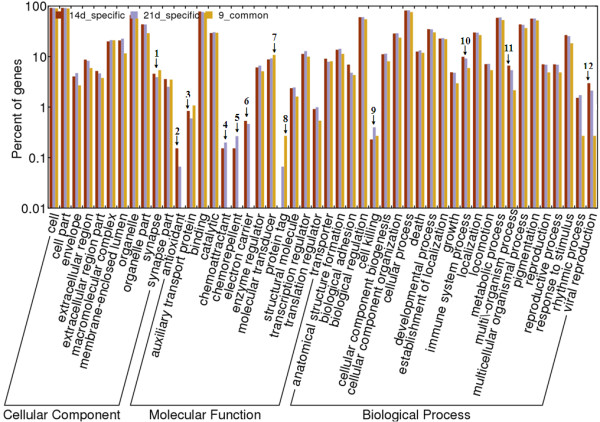
**Enrichment analysis for the targets of miRNAs in the brains of *Toxoplasma gondii*-infected mice 14 and 21 days postinfection.** Annotated numbers were used to mark columns that had different percentages in different samples.

For global analysis, the GO items of the targets were enrichment-analyzed (Figure 2). For the 9 miRNAs common to both time points post-infection, the numbers of different GO items represented were generally lower than those of the 14d-specific and 21d-specific miRNAs at all of the 3 classifications in GO analysis (cellular component, molecular function and biological process). For cellular component, only “synapse” had a marginally higher percentage in the common miRNAs than in the 14d- or 21d-specific miRNAs (Figure 2, annotated with “1”), however, there was a significantly higher percentage of the common, rather than the post-infection time point specific miRNAs focused on the “protein tag” function (Figure 2, annotated with “8”). In addition, a further two functions (“auxiliary transport protein” and “molecular transducer”) were also represented marginally higher in the common, rather than the post-infection time point specific miRNAs (Figure 2, annotated with ″3‶ and ″7‶).

Targets of the 14d-specific and 21d-specific miRNAs contained 4 functions, which were not identified in the 9 common miRNAs; “antioxidant”, “chemoattractant”, “chemorepellent” and “electron carrier” (Figure 2, annotated with ″2‶, ″4‶, ″5‶, and ″6‶). Targets of the 14d-specific miRNAs had significantly higher representation in the “antioxidant” function than those of the 21d-specific miRNAs (Figure 2, annotated with “2”) and marginally higher representation in the “electron carrier” function (Figure 2, annotated with “6”). Targets of the 21d-specific miRNAs were relatively more abundant in the “chemoattractant” and “chemorepellent” functions (Figure 2, annotated with ″4‶ and ″5‶), and “protein tag” functions (Figure 2, annotated with “8”). For biological process, the targets of 14d-specific miRNAs had marginally higher percentages in “immune system process”, “multi-organismal process” and “viral reproduction” processes than either the common miRNAs or the 21d-infected miRNAs (Figure 2, annotated with ″10‶, ″11‶ and ″12‶), but targets of the 21d-specific miRNAs had significantly higher representation in the ‶cell killing” process (Figure 2, annotated with ″9‶).

Among the targets, some immune-related functionality was found, including “Chemokine”, “Cytokine”, “Growth factor” and “Interleukin”. For chemokine-related targets/factors/receptors, there were 12 targets associated with 14d-specific miRNAs, including “binding activity” (GO:0031726) and “antagonist activity” (GO:0046817), but only one target (GO:0008009) associated with 21d-specific miRNAs and the 9 miRNAs common to both time points post-infection. For cytokine-related factors, 3 and 9 targets were associated with the 9 miRNAs common to both time points post-infection and 14d-specific miRNAs respectively, while there were 18 targets associated with the 21d-specific miRNAs. For “growth factor”, there were 12, 57 and 66 targets respectively for the 9 miRNAs common to both time points post-infection, 14d-specific and 21d-specific miRNAs; including fibroblast growth factor (GO:0005105), epidermal growth factor (GO:0005154), nerve growth factor (GO:0048406) and vascular endothelial growth factor (GO:0005021). For “interleukins” (IL), receptors of IL-3, IL-6, and IL-23 were amongst the targets of the 9 miRNAs common to both time points post-infection; receptors of IL-1 and IL-7 were associated with the 14d-specific miRNAs and the majority of the interleukin receptors were amongst the targets of the 21d-specific miRNAs, including IL-1, IL-2, IL-3, IL-7 and IL-23.

### miRNA quantification

**Figure 3:**
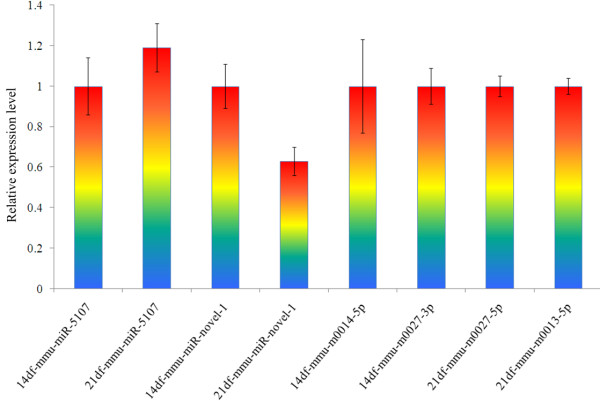
Quantitative PCR analysis of the 6 representative miRNAs.

Six representative miRNAs, including mmu-mir-5107 and mmu-miR-novel-1 (common to both time points post-infection); 14df-m0014-5p and 14df-m0027-3p (14d-specific miRNAs), and 21df-m0027-5p and 21df-m0013-5p (21d-specific miRNAs), which had higher copy numbers than others, were chosen and used in a quantitative PCR analysis (Figure 3). All 6 miRNAs were successfully amplified. The mmu-miR-5107 was expressed marginally higher in the 21d sample (1.19 ± 0.12) than in the 14d (1 ± 0.14), while the expression level of mmu-miR-novel-1 was slightly lower in the 21d sample (0.63 ± 0.07) than that in the 14d RNA (1 ± 0.11). The other 4 miRNAs were at nearly same level as the endogenous control with expression levels of 1 ± 0.23 (14df-mmu-m0014-5p), 1 ± 0.09 (14df-mmu-m0027-3p), 1 ± 0.05 (21df-mmu-m0027-5p), and 1 ± 0.04 (21df-mmu-m0013-5p).

## Discussion

Invasion and survival of parasitic protozoa in a hostile host environment requires multiple gene expression adaptations for modifying or manipulating the host environment, some of which are thought to involve parasite-derived miRNAs [30]. In addition, host-derived miRNAs control host protein production; for example human miRNAs are reported to control the expression of around 60% of human protein-coding genes and, because of these key regulatory functions, deregulation of miRNAs has been associated with the pathogenesis, progression and prognosis of different diseases [31]. To analyze the interplay between parasite and host at the miRNA levels, we investigated the changes in miRNA profiles of mouse brains 14 and 21 d after infection with cyst-forming *T. gondii*, which had more cyst burdens than other time points [32].

Total numbers of miRNAs identified were similar (~500) in infected brain samples and controls, and most of these (414) were conserved in both infected and uninfected brain samples, regardless of the time points post infection, indicating fundamental regulatory functions of most of these miRNAs. In addition, multiple time and infection status-specific miRNAs were also found indicating that the miRNA expression profiles changed over time, whether parasites were present or not. For the 14d sham-infected control, there were 35 specific miRNAs which were not detected following *T. gondii* invasion but were replaced by the 17 “14d infection-specific” miRNAs. In a recent study [19], it was found that *T. gondii* infection changed the expression level of miR-17 (approximately 92 times) and miR-106b (approximately 25 times) in cultured HFFs, although the mechanisms driving their altered expression are unknown. These miRNAs were supposed to regulate development and metabolism of host cells. During the invasion of host cells by the parasite, some metabolism pathways were closed and redirected, and new pathways conducted by the parasite were generated as well, therefore, old miRNAs were replaced by new ones. A similar phenomenon was also found in the mouse brains at 21d post-infection in the present study. Therefore, *T. gondii* invasion altered the host miRNA expression, in agreement with the results of Zeiner *et al.*[19], although there were many factors, which are difficult to be well controlled, such as the host condition, different stress factors and the virulence of the parasite, which may influence the results.

The 21d infected sample contained miRNAs whose predicted targets possessed “protein tag” functions – a function which was absent from the targets of the miRNAs derived from the 14d-specific sample (Figure 2). In addition “protein tag” targets were also found in the 9 miRNAs common to both time points post-infection. This indicates that miRNAs for which targets have “protein tag” functions were present after 14d post infection and further enhanced by 21d post infection. *Toxoplasma gondii* can change host metabolism and reproduce itself alongside host processes and factors [9,12], therefore, correct protein tags are required for the host to identify “self” proteins and guide them into the appropriate metabolic pathways, which may be a competitive mechanism for dealing with parasitism. In connection with this, it has been reported that the secreted proteins of the parasite may be undergoing accelerated evolution, possibly as a response to stronger selective pressure from host [30]. The “protein tag” function of the host miRNA targets may thus represent a host anti-invasion strategy in response to such accelerated evolution of the parasite.

Immune-related targets including chemokines, cytokines, growth factors and interleukins were also found. These factors all have roles in the host immune response to parasite challenge: chemokines and their receptors are important in the control of parasite replication and acute cardiac inflammation, and trigger proinflammatory responses to many microbial pathogens [33,34]. At the time point of 14d post infection more chemokine targets were identified than at 21d post infection, with 12 chemokine-related targets in the 14d sample and only 1 in the 21d sample. In contrast, the numbers of cytokine, growth factor and interleukin targets were much higher in the 21d sample than in the 14d sample. Interleukins have been reported as being important host protection factors against parasites, such as gastrointestinal nematodes and protozoan parasites [35,36] and, from the data presented here, the roles of interleukin targets as well as those for cytokines and growth factors increase with the development of *T. gondii* infections.

## Conclusions

The present study characterized the expression profiles of miRNA in the brain of mice during the invasion of *T. gondii*. This analysis showed that the miRNA expression of the host brain can be altered by the invasion of cyst-forming *T. gondii*, and that the host might attempt to protect itself by marking proteins with “protein tags” and through the increased regulation of immune related systems.

## Competing interests

The authors declare that they have no competing interests.

## Authors’ contributions

XQZ conceived and designed the study, and critically revised the manuscript. MJX and DHZ performed the experiments, analyzed the data and drafted the manuscript. AJN, SYH and YFF helped in study design, study implementation and manuscript revision. All authors read and approved the final manuscript.

## Supplementary Material

Additional file 1: Table S1 Common and specific miRNAs of *Toxoplasma gondii* infected mouse brain at 14 d and 21d post infection. Click here for file
