# Supplementary material for: Characterization of mouse brain microRNAs after infection with cyst-forming Toxoplasma gondii
Source: Parasit Vectors. 2013 May 29;6:154. doi: 10.1186/1756-3305-6-154 (PMC3668261; doi:10.1186/1756-3305-6-154)
Supplement: Additional file 1: Table S1 — Common and specific miRNAs of Toxoplasma gondii infected mouse brain at 14 d and 21d post infection. [file 1756-3305-6-154-S1.docx]

**Table S1. Common and specific miRNAs of *Toxoplasma gondii* infected mouse brain at 14 d and 21d post infection.**

| **Name** | **Target number** | **Best matched target** | **Gene ID** | **Target description** |
| --- | --- | --- | --- | --- |
| **9_common miRNA** | |  |  |  |
| mmu-miR-5107 | 212 | Pafah2 | NM_133880 | platelet-activating factor acetylhydrolase 2 |
| mmu-miR-5133 | 143 | Pdcd7 | NM_016688 | programmed cell death 7 |
| mmu-miR-211 | 69 | Ddx19b | NM_001190786 | DEAD (Asp-Glu-Ala-Asp) box polypeptide 19b |
| mmu-miR-3963 | 34 | Pten | NM_008960 | phosphatase and tensin homolog |
| mmu-miR-147 | 16 | Ammecr1 | NM_019496 | Alport syndrome, mental retardation, midface hypoplasia and elliptocytosis chromosomal region gene 1 homolog (human) |
| mmu-miR-1895 | 11 | Wdr89 | NM_028203 | WD repeat domain 89 |
| mmu-miR-nov-com-2 | 14 | Gm19784 | NR_040461.1 | *Mus musculus* predicted gene, 19784 |
| mmu-miR-nov-com-1 | 2 | D930015E06 | NM_172681.4 | *Mus musculus* RIKEN cDNA D930015E06 gene |
|  |  |  |  |  |
| **14d_infected specific miRNA** | |  |  |  |
| mmu-miR-9 | 1076 | KEL | NM_000420 | Kell blood group, metallo-endopeptidase |
| mmu-miR-470 | 203 | ACER3 | NM_018367 | alkaline ceramidase 3 |
| mmu-miR-3105-5p | 176 | ZNF664 | NM_001204298 | zinc finger protein 664 |
| mmu-miR-290-5p | 166 | SAMD5 | NM_001030060 | sterile alpha motif domain containing 5 |
| mmu-miR-546 | 153 | SHARPIN | NM_030974 | SHANK-associated RH domain interactor |
| mmu-miR-1936 | 122 | CREBZF | NM_001039618 | CREB/ATF bZIP transcription factor |
| mmu-miR-5103 | 7 | FOXP4 | NM_001012426 | forkhead box P4 |
| mmu-miR-1932 | 5 | ANKRD52 | NM_173595 | ankyrin repeat domain 52 |
| mmu-miR-5126 | 4 | CAMK2G | NM_001204492 | calcium/calmodulin-dependent protein kinase II gamma |
| mmu-miR-nov-14d-speci-1 | 8 | DXBay18 | NM_001025384.3 | *Mus musculus* DNA segment, Chr X, Baylor 18 |
| mmu-miR-nov-14d-speci-2 | 3 | septin | NM_027669.3 | *Mus musculus* septin 12 |
| mmu-miR-nov-14d-speci-3 | 6 | Srsf11 | NM_001093753.1 | *Mus musculus* serine/arginine-rich splicing factor 11 |
| mmu-miR-nov-14d-speci-4 | 3 | Nid1 | NM_010917.2 | *Mus musculus* nidogen 1 |
| mmu-miR-nov-14d-speci-5 | 8 | Cd2bp2 | NM_027353.3 | *Mus musculus* CD2 antigen |
| mmu-miR-nov-14d-speci-6 | 4 | Ano9 | NM_178381.3 | *Mus musculus* anoctamin 9 |
| mmu-miR-nov-14d-speci-7 | 3 | 1700054N08Rik | NM_028536.1 | *Mus musculus* RIKEN cDNA 1700054N08 gene |
| mmu-miR-nov-14d-speci-8 | 7 | Ndst2 | NM_010811.2 | *Mus musculus* N-deacetylase/N-sulfotransferase |
|  |  |  |  |  |
| **21d_infected specific miRNA** | |  |  |  |
| mmu-miR-694 | 888 | CHCHD1 | NM_203298 | coiled-coil-helix-coiled-coil-helix domain containing 1 |
| mmu-miR-5101 | 435 | C18orf34 | NM_001105528 | chromosome 18 open reading frame 34 |
| mmu-miR-1955-5p | 191 | SLC35B4 | NM_032826 | solute carrier family 35, member B4 |
| mmu-miR-1196 | 174 | BCL2L11 | NM_001204106 | BCL2-like 11 (apoptosis facilitator) |
| mmu-miR-18a | 173 | IGSF3 | NM_001007237 | immunoglobulin superfamily, member 3 |
| mmu-miR-599 | 120 | BNC2 | NM_017637 | basonuclin 2 |
| mmu-miR-741 | 98 | MTDH | NM_178812 | metadherin |
| mmu-miR-875-5p | 85 | BCL11A | NM_022893 | B-cell CLL/lymphoma 11A (zinc finger protein) |
| mmu-miR-3544 | 81 | PDCD7 | NM_005707 | programmed cell death 7 |
| mmu-miR-3091-5p | 40 | LPP | NM_001167671 | LIM domain containing preferred translocation partner in lipoma |
| mmu-miR-18b | 22 | JMY | NM_152405 | junction mediating and regulatory protein, p53 cofactor |
| mmu-miR-466f | 15 | LPP | NM_001167671 | LIM domain containing preferred translocation partner in lipoma |
| mmu-miR-719 | 7 | DAB2IP | NM_032552 | DAB2 interacting protein |
| mmu-miR-nov-21d-speci-1 | 5 | Cacna2d2 | NM_020263.3 | *Mus musculus* calcium channel, voltage-dependent, alpha 2/delta subunit 2 |
| mmu-miR-nov-21d-speci-2 | 3 | Slc5a5 | NM_053248.2 | *Mus musculus* solute carrier family 5 |
| mmu-miR-nov-21d-speci-3 | 7 | Cttnbp2 | NM_080285.1 | *Mus musculus* cortactin binding protein 2 |
| mmu-miR-nov-21d-speci-4 | 4 | Eef2k | NM_001267711.1 | *Mus musculus* eukaryotic elongation factor-2 kinase |
| mmu-miR-nov-21d-speci-6 | 5 | Lck | NM_001162433.1 | *Mus musculus* lymphocyte protein tyrosine kinase |
| mmu-miR-nov-21d-speci-8 | 2 | Tpm2 | NM_009416.3 | *Mus musculus* tropomyosin 2, beta |
| mmu-miR-nov-21d-speci-9 | 2 | Adcy7 | NM_001109756.1 | *Mus musculus* adenylate cyclase 7 |
| mmu-miR-nov-21d-speci-10 | 2 | Ptpn2 | NM_001127177.1 | *Mus musculus* protein tyrosine phosphatase, non-receptor type 2 |
| mmu-miR-nov-21d-speci-11 | 11 | A130010J15Rik | NM_001160359.1 | *Mus musculus* RIKEN cDNA A130010J15 gene |
| mmu-miR-nov-21d-speci-12 | 5 | St3gal1 | NM_009177.4 | *Mus musculus* ST3 beta-galactoside alpha-2,3-sialyltransferase 1 |
| mmu-miR-nov-21d-speci-13 | 3 | Elmo3 | NM_172760.3 | *Mus musculus* engulfment and cell motility 3 |
| mmu-miR-nov-21d-speci-14 | 1 | Nr6a1 | NM_001159549.1 | *Mus musculus* nuclear receptor subfamily 6, group A, member 1 |
| mmu-miR-nov-21d-speci-15 | 6 | Nt5dc3 | NM_175331.3 | *Mus musculus* 5'-nucleotidase domain containing 3 |
| mmu-miR-nov-21d-speci-16 | 2 | Stx11 | NM_001163591.1 | *Mus musculus* syntaxin 11 |
| mmu-miR-nov-21d-speci-17 | 3 | Lipm | NM_023903.1 | *Mus musculus* lipase, family member M |
| mmu-miR-nov-21d-speci-18 | 5 | Plec | NM_201394.2 | *Mus musculus* plectin |
| mmu-miR-nov-21d-speci-19 | 10 | Pak7 | NM_172858.2 | *Mus musculus* p21 protein |
| mmu-miR-nov-21d-speci-20 | 2 | Clcn3 | NM_173873.1 | *Mus musculus* chloride channel 3 |
